# The Map in Our Head Is Not Oriented North: Evidence from a Real-World Environment

**DOI:** 10.1371/journal.pone.0135803

**Published:** 2015-09-09

**Authors:** Tad T. Brunyé, Heather Burte, Lindsay A. Houck, Holly A. Taylor

**Affiliations:** 1 Center for Applied Brain & Cognitive Sciences, Medford, Massachusetts, United States of America; 2 Department of Psychology, Tufts University, Medford, Massachusetts, United States of America; 3 Cognitive Science Team, U.S. Army Natick Soldier Research, Development and Engineering Center, Natick, Massachusetts, United States of America; University of Sussex, UNITED KINGDOM

## Abstract

Like most physical maps, recent research has suggested that cognitive maps of familiar environments may have a north-up orientation. We demonstrate that north orientation is not a necessary feature of cognitive maps and instead may arise due to coincidental alignment between cardinal directions and the built and natural environment. Experiment 1 demonstrated that pedestrians have difficulty pointing north while navigating a familiar real-world environment with roads, buildings, and green spaces oriented oblique to cardinal axes. Instead, north estimates tended to be parallel or perpendicular to roads. In Experiment 2, participants did not demonstrate privileged memory access when oriented toward north while making relative direction judgments. Instead, retrieval was fastest and most accurate when orientations were aligned with roads. In sum, cognitive maps are not always oriented north. Rather, in some real-world environments they can be oriented with respect to environment-specific features, serving as convenient reference systems for organizing and using spatial memory.

## Introduction

Understanding how individuals acquire, represent, and use spatial knowledge is fundamental to predicting and optimizing navigation behavior. Tolman’s early [1] proposal that mammals embody map-like spatial memories is both intuitive and controversial. Under this theory, humans construct map-like memories of familiar environments that preserve information about inter-landmark relationships and Euclidean distances. Though the cognitive map metaphor has been repeatedly challenged [2–4] some recent data suggest its explanatory power in understanding the organization of spatial memory. For instance, Gagnon and colleagues demonstrated that environments are more easily learned when initial path segments went northward, aligning oneself with emergent cardinal axes and top-down perspective of a cognitive map [5]. Interestingly, this pattern emerges even without exposure to a real map of the environment, suggesting that cognitive maps default to a north orientation. Similarly, Frankenstein and colleagues [6] demonstrated privileged access to spatial memories while participants were oriented toward north in a virtual environment. Together, these studies suggest that spatial memory for familiar environments is related to cardinal direction, much like a cartographer’s map.

The present paper challenges the notion that cognitive maps are necessarily oriented toward the north. Instead, we suggest that navigators assign a spatial reference system based on salient and functional environmental features, such as roads, rivers, and landmarks [7,8]. Critically, the natural alignment or misalignment of these features with cardinal axes dictates the apparent organization of cognitive maps. Specifically, when environmental features are aligned with cardinal axes, cognitive maps can develop serendipitous alignment with north. Indeed the environments used in prior work demonstrate natural or built alignment with cardinal directions. In [5] the environments contained roads and large scale features oriented parallel and perpendicular with cardinal axes, and in [6] the environment includes a river running east-west through the town. Confounding cardinal axes and environmental features makes it difficult to dissociate their organizational influence on cognitive maps.

In contrast to environments tested in prior studies, many real-world environments contain large-scale features misaligned (oblique) with cardinal axes. For instance, Santa Barbara, CA has a downtown road network arranged in a grid but oriented approximately 48° oblique to true north, Manhattan New York, NY is oriented 29° oblique to true north, and Pittsburgh, PA has a road network and three intersecting rivers also arranged obliquely relative to cardinal axes. In these cases, it is unclear whether cognitive maps would be oriented toward north given that large-scale environmental features are oblique to cardinal axes. For the present study we focused on the Tufts University (Medford, MA) campus, which is characterized by road, building, and green spaces oriented 42° oblique to magnetic north (currently 27° oblique to true north). This environment presents the opportunity to assess whether the alignment of cognitive maps with cardinal axes is a necessary organizational principle or simply arises in some environments due to correlations between environmental features and cardinal directions.

## Experiment 1

In our first experiment, we asked pedestrians navigating this environment to spontaneously point toward north. If cognitive maps of familiar environments are indeed oriented north, then pedestrians should be able to accurately point in this direction. In contrast, if pedestrians show high error rates in their pointing estimates, then it is unlikely their cognitive maps are organized with reference to cardinal axes. Instead, they may show evidence for referencing estimates relative to environmental features such as roads [8,9], or no evidence of feature reliance or north awareness whatsoever [10]

### Method

#### Ethical Compliance

Research was approved by the Tufts University Social, Behavioral, and Educational Research Institutional Review Board (SBER IRB; protocol 1310019). This experiment was approved under provisions of Exempt Category 2 as defined in 45 CFR 46.101 (b), and thus participants did not provide consent.

#### Participants & Design

Two hundred Tufts University students (*M*_age_ = 19.7, *SD*_age_ = 1.3, 92 male & 108 female) with varied experience living on campus (*M* = 1.8 years, *Range* = 0 to 4 years). This desired sample size was based on prior research using pointing tasks in outdoor environments, which also used 20 participants per location [9]. This was a field study with no manipulated variables.

#### Materials

**Fig 1:**
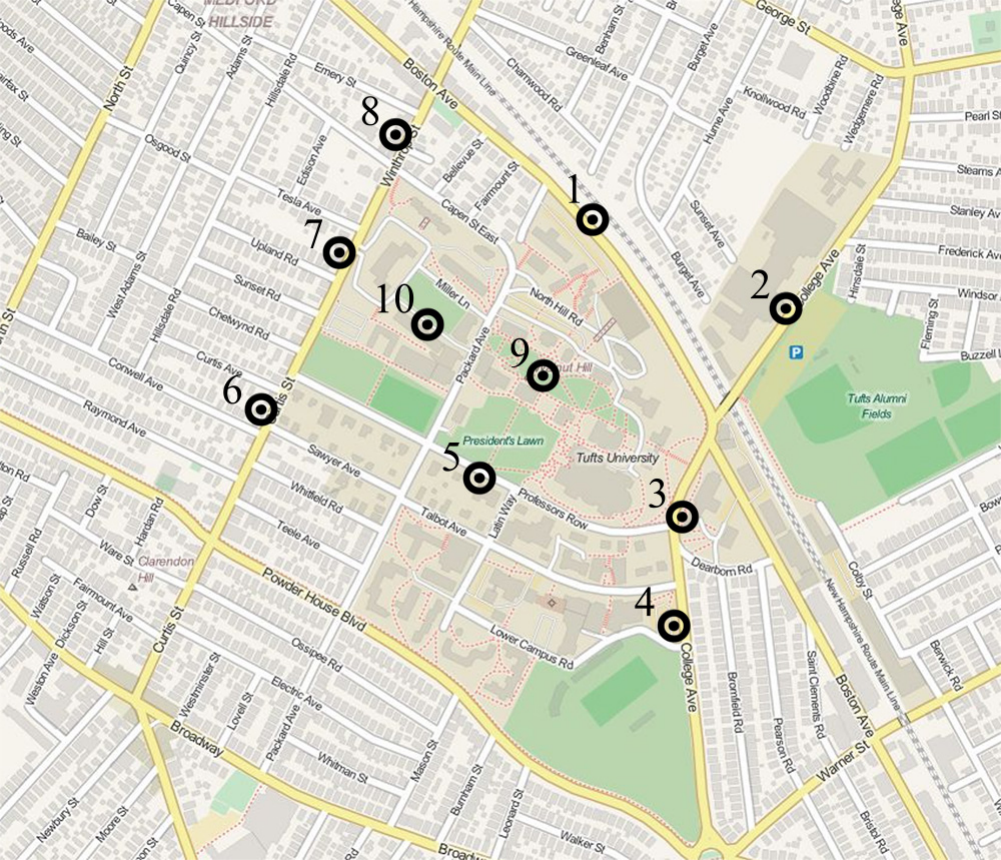
Data collection sites overlaid on the Tufts University campus map (with a true north orientation). Map generated using OpenStreetMap, shared under the CC BY-SA license. http://www.openstreetmap.org/copyright.

Ten data collection locations were selected on the Tufts campus; all were immediately adjacent to a road (but not an intersection), and situated on a sidewalk with moderate pedestrian traffic. Together the locations sampled the central and peripheral campus (see Fig 1 for location details). We used a Suunto A-30L Field Compass to measure participant pointing responses. A demographics form probed for age, sex, and number of years living on or near campus. We also used the Santa Barbara Sense of Direction (SBSOD), a set of 15 questions probing self-reported environmental spatial ability [11].

### Procedures

At each data collection site, two experimenters solicited pedestrian participants; all were required to be Tufts students. Participants were asked to point north and an experimenter recorded pointing angles relative to magnetic north. Participants then completed the demographics form and SBSOD. For the SBSOD, questions are answered on scales anchored at 1 (*Strongly Agree*) and 7 (*Strongly Disagree*), negatively phrased items are reverse-scored, and an average score was calculated. After completing the pointing task and questionnaires, participants were compensated $5 for their time (approximately 10 minutes).

#### Data Scoring

To score the pointing estimate data, we related the pointing vector of the participant’s arm to magnetic North. To measure adjacent route orientation, for each of the 10 data collection locations we plotted a vector along 200m of the adjacent route using Google Earth and recorded its orientation relative to North. Because our compass measured magnetic north, and road orientations on Google Earth are depicted with reference to true north, we converted all Google Earth outputs to magnetic north with reference to the average declination over the course of the data collection effort (14.8° W centered on 42.407508° N, 71.119142° W). To do so, we used the International Geomagnetic Reference Field (IGRF) model (http://www.ngdc.noaa.gov/geomag-web/#declination). To test for overall accuracy, we used circular statistics to calculate mean [12] pointing angle. Data are included in S1 Dataset.

### Results

#### Overall Accuracy

Collapsed across the 10 data collection locations, participants showed a mean angle of 19.57° from magnetic north (calculated using circular statistics; mean vector *r* = .43). A one-sample circular mean angle test [13] demonstrated that this mean pointing angle was significantly different from 0 (magnetic north).

A Rayleigh’s circular *z* test demonstrated that pointing was not uniformly distributed (Rayleigh’s *z* = 37.5, *p* < .001). The majority of participants (n = 151/200) showed estimates within the 180° semi-circle centered on north, and this exceeded that of chance (χ^2^ (1) = 52.02, *p* < .001). In other words, participants showed only coarse knowledge of cardinal direction within a 180° region.

#### Individual Differences

We evaluated whether participant sex, years of experience with the campus, or sense of direction (*M* = 4.12, range 1.47–6.8) would predict overall pointing error. A multiple linear regression was conducted, with three predictors: sex, years of experience with the campus, and sense of direction (SBSOD). The single dependent variable was absolute angular pointing error (0° to 180°). The overall regression model was significant, *F*(4, 199) = 8.37, *p* < .001, *R* ^2^ = .11. Only one predictor, SBSOD, reached significance, *t*(199) = 3.78, *p* < .001, β_std_ = -.26; higher spatial sense of direction scores predicted lower pointing error.

#### Follow-up Analysis

**Fig 2:**
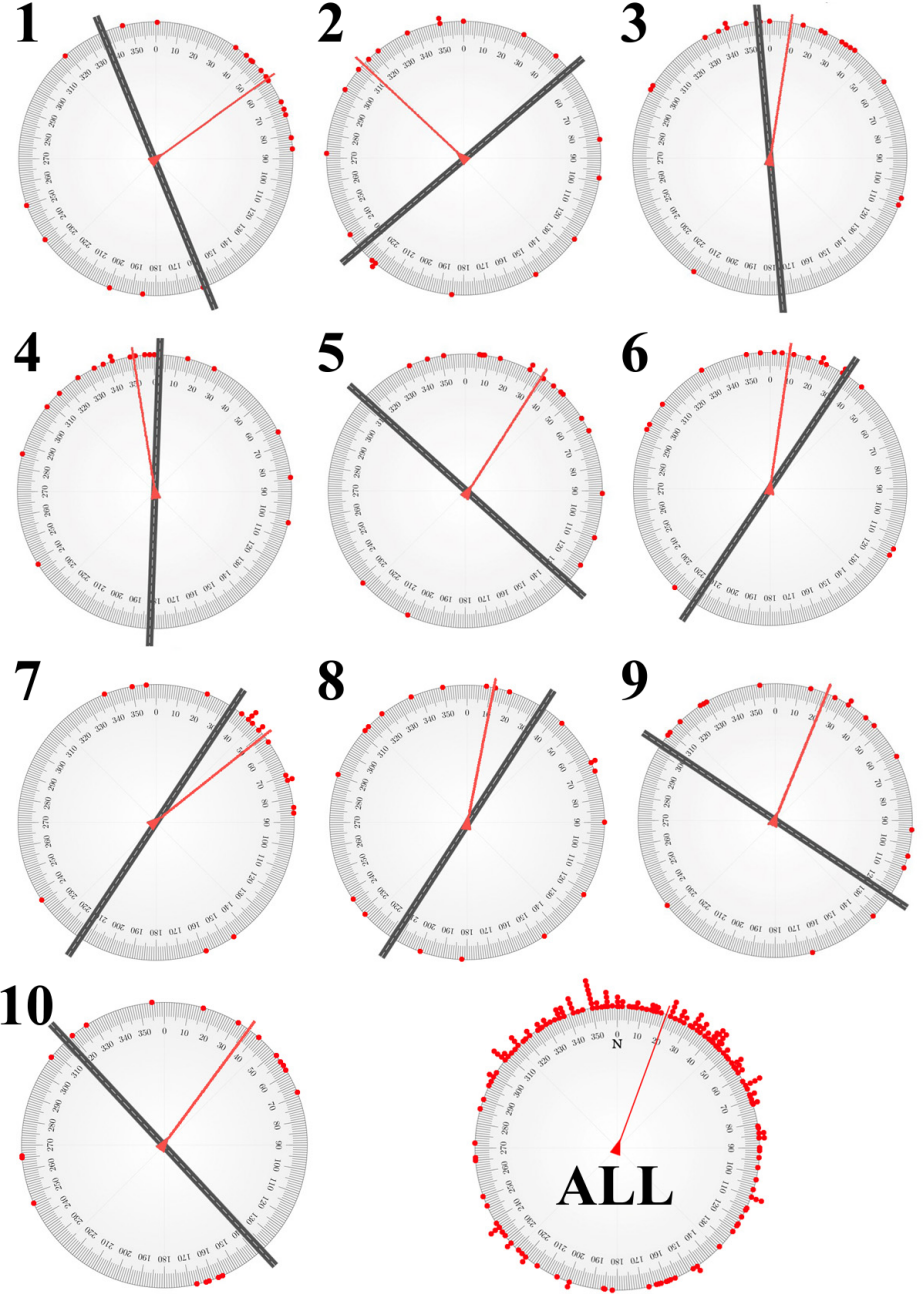
Pointing estimates for all locations (ALL) and each of the 10 data collection locations overlaid onto a magnetic compass, with adjacent road orientation and mean pointing direction indicated (using circular statistics). Note that this figure is aligned with magnetic north, whereas Fig 1 is aligned with true north; relating the two figures must thus consider declination.

The pointing estimates at each of the 10 campus locations, with reference to the adjacent road orientation, show a striking pattern: mean pointing angles were consistently related to road orientations. Fig 2 depicts this pattern by using circular statistics [12] to calculate mean pointing directions. Five locations showed mean pointing angles roughly parallel to adjacent road orientation, and the other five showed mean pointing angles roughly perpendicular to adjacent road orientation. In other words, on average, participants referenced their pointing estimates to nearby road orientations, aligning their north estimates either parallel or perpendicular to the nearest road. We tested this pattern by conducting one-sample circular mean angle tests at each location, asking whether pointing angles differ from the angle parallel (locations 3, 4, 6, 7, 8) or perpendicular (locations 1, 2, 5, 9, 10) to adjacent roads. In no location did the pointing angles differ significantly from parallel or perpendicular to adjacent roads (α = .05).

### Experiment 1 Discussion

Our first experiment demonstrates only coarse awareness of cardinal directions in pedestrians navigating a familiar large-scale environment. Even individuals who were highly familiar with the campus environment showed overall poor awareness of cardinal directions. This pattern was qualified by those with a high sense of direction tending to show higher accuracy, converging with research suggesting the sense of direction questionnaire indexes trait-based differences in the ability to orient oneself with regard to global spatial reference systems [11]. The predictive value of sense of direction on pointing accuracy was rather weak, however.

## Experiment 2

Results of our first experiment provide some suggestion that pedestrians show difficulty orienting themselves with regard to cardinal direction. In an environment that is oriented oblique to cardinal axes, participants instead relied on road orientations to guide pointing estimates. Road networks may structure cognitive maps in some environments. It could also be the case that pedestrians are only relying on road orientations because they are of high functional relevance to their navigation task, shifting reliance on perceptible features versus spatial memory due to task demands [14]. Experiment 2 thus tests spatial memory retrieval in an indoor, seated context, using a judgment of relative direction (JRD) task.

JRD tasks are able to assess the influence of alignment with spatial reference systems by orienting participants in particular directions (e.g., *Standing at the Library and facing Olin Hall*) and then testing their memory for the direction of a third landmark (e.g., *Point to the Tennis Courts*). We refer to the initial orientation as the *default vector*, and the pointed to location as the *target landmark*. A large body of literature demonstrates that aligning the default vector with a spatial reference system facilitates pointing accuracy and efficiency [7,15–17]. If participants rely on cardinal axes as a spatial reference system [6], they should show faster and more accurate responses when the default vector is aligned with cardinal axes. In contrast, if participants rely on road orientations (as suggested by Experiment 1 and [8,9,18]), they should show faster and more accurate responses when a default vector is aligned parallel or perpendicular to primary roads.

### Method

#### Ethical Compliance

Research was approved by the Tufts University Social, Behavioral, and Educational Research Institutional Review Board (SBER IRB; protocol #1310028), with secondary approvals from the funding agency (U.S. Army Human Research Protections Office). Participants provided written informed consent to participate in the study.

#### Participants & Method

Forty-four Tufts University students (*M*_age_ = 19.8, *SD*_age_ = 1.3, 17 male & 27 female) with varied years of experience living on campus (*M* = 1.82 years, *Range* = 0 to 4 years) participated. Our intent was to collect 36 complete data sets (after excluding due to unawareness of particular landmarks, see below). This desired sample size was based on prior work using judgment of relative direction tasks [19]. Each participant completed a series of questionnaires than completed a JRD task; in a within-participants design, JRD trials varied the default vector between cardinal, road-oriented, and arbitrary orientations.

#### Materials

The same demographic form used in Experiment 1 probed for age, sex, and number of years living on or near campus, and we also administered the SBSOD.

We developed a judgment of relative direction (JRD) task with 48 trials. The task used 12 landmarks from the Tufts campus that, based on our earlier research, tend to be most frequently recalled during map drawing tasks. Each trial used 3 of these landmarks: two to define a default vector (e.g., *Standing at* ***Miller Hall***, *facing* ***Barnum Hall***) and one to define a target landmark (e.g., *Point to* ***Eaton Hall***). Four trial conditions were developed, each with default vectors oriented parallel to: magnetic cardinal axes (12 trials), true cardinal axes (12 trials), major campus road axes (16 trials), or oblique to both cardinal and road axes (8 control trials). In each condition, initial vectors were balanced across orientations (e.g., N to S, S to N, E to W, W to E), and correct pointing directions were both positive (clockwise) and negative (counter-clockwise) angles relative to the initial vector, and distributed around the entire 360° range. We also took care to promote similar overall initial vector lengths in the cardinal (true, magnetic) and road axis conditions, as demonstrated in a confirmatory one-way ANOVA, *F*(2, 39) = .13, *p* = .88, η^2^ < .01.

### Procedures

Participants provided informed consent and then completed the two questionnaires. They then completed the 48 JRD task trials. A custom software package was developed using the Microsoft C# programming language to display JRD trials and collect data. On each trial the default vector was vertically aligned on the monitor with the *standing at* and *facing* landmarks depicted as labeled black dots 245 pixels apart. An arrow 130 pixels in length emanated from the *standing at* landmark and pointed toward the *facing* landmark. When mouse-clicked, the arrow would rotate around while its base remained anchored on the *standing at* landmark. At the top of the screen the *point to* landmark was verbally provided. The 48 trials were presented one at a time in the center of the screen in random order, and participants were provided with unlimited time to response to each trial. To respond, participants would click on the arrow and rotate it around until it pointed in their preferred direction. When they were satisfied with the pointing direction, they would click the next button to continue. For each trial, the software outputted signed (positive clockwise, negative counter-clockwise) angular deviation from the correct pointing direction to the indicated position, and response time. Completing the 48 trials took approximately 10 minutes. An additional questionnaire asked participants to specify if they did not know the location of any of the 12 Tufts JRD landmarks. Note that participants were always seated at a computer facing a direction (true heading 240°) oblique to both cardinal directions and primary campus road axes. Participants were compensated $10 for their time (approximately 20 minutes).

### Data Scoring & Analysis

Eight participants, who did not know at least one landmark location as indicated on the final questionnaire, were eliminated from analyses, resulting in 36 complete data sets. Because we made no hypotheses regarding directional (right/left) biases, we analyzed pointing error using absolute angular error values. For response times, we log_10_-transformed the data to correct for a positive skew (Fisher’s skewness pre = 2.15, post = .45); pre-transformed means are detailed in Table 1. Two repeated-measures ANOVAs were conducted, one for angular error and one for response times; the ANOVAs considered the 4 alignment conditions of interest: aligned with magnetic cardinal axes, true cardinal axes, major campus road axes, or oblique to both cardinal and road axes. Follow-up comparisons used paired samples t-tests with a Bonferroni corrected alpha (α = .017). As in Experiment 1, we also assessed whether individual differences in participant sex, years on campus, and sense of direction modulated any of these effects. Data are included in S2 Dataset.

**Table 1:** Raw (pre-transform) response time means and standard deviations for each of the four alignment conditions.

	Magnetic Axes	True Axes	Road Axes	Off-Axis
Mean (msec)	17287.1	15976.7	14157.1	14318.8
SD	7971.9	8056.1	5975.5	4686.2

### Results

#### Angular Error

**Fig 3:**
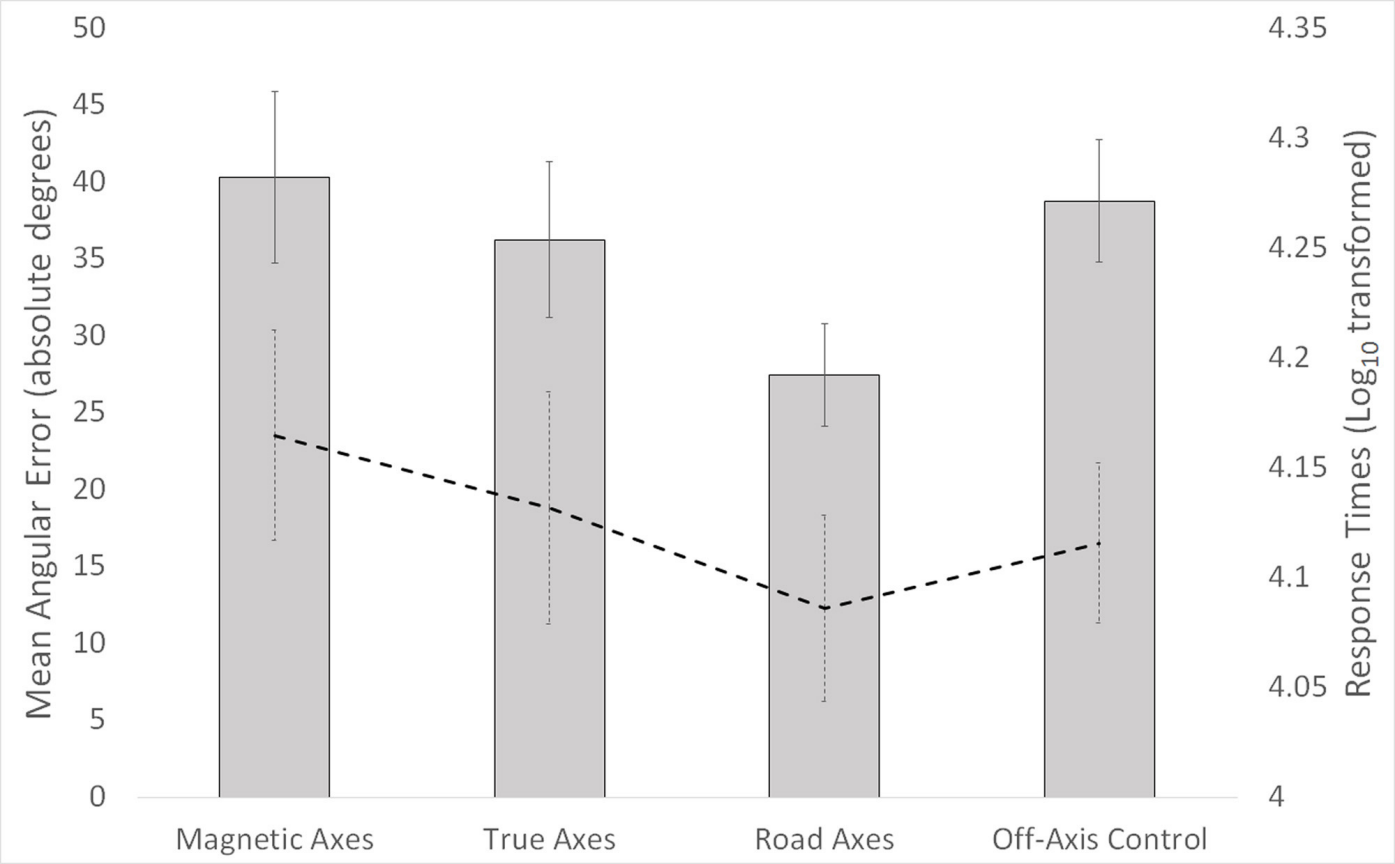
Bars depict mean angular error, in absolute degrees, for each of the four alignment conditions. Line depicts mean response times (log_10_-transformed), for each of the four alignment conditions. Error bars indicate 95% confidence intervals.

A main effect of alignment, F(3, 105) = 11.57, p < .001, η2 = .25, demonstrated lowest angular error in the road aligned condition relative to the other three conditions, as depicted in Fig 3. Paired t-tests confirmed lower error in the road aligned condition relative to the magnetic aligned, t(35) = 5.39, p < .001, Cohen’s d = .90, true aligned, t(35) = 3.89, p < .001, Cohen’s d = .65, and off-axis control, t(35) = 5.98, p < .001, Cohen’s d = 1.0, conditions.

#### Response Times

A main effect of alignment, F(3, 105) = 4.53, p < .01, η2 = .12, demonstrated fastest response times in the road aligned condition relative to the other three conditions, as detailed in Fig 3. Paired t-tests confirmed faster response times in the road aligned condition relative to the magnetic aligned, t(35) = 5.34, p < .001, Cohen’s d = .90, and true aligned, t(35) = 2.84, p < .01, Cohen’s d = .49, conditions. The road aligned and off-axis control conditions did not differ, t(35) = 1.27, p = .21.

#### Individual Differences

A series of linear multiple regressions assessed whether participant sex, years of experience with the campus, or sense of direction (*M* = 3.59, range 2.33–5.4) predicted angular error in each of the four alignment conditions. Two regressions reached significance. First, the regression predicting angular error in the road-aligned condition, *F*(3, 35) = 2.88, *p* = .051, *R* ^*2*^ = .21, with higher SBSOD scores predicting lower error, *t*(35) = 2.38, *p* = .024, β_std_ = .38. Second the regression predicting angular error in the off-axis control condition, *F*(3, 35) = 3.12, *p* < .05, *R* ^*2*^ = .23, with higher SBSOD scores predicting lower error, *t*(35) = 3.05, *p* < .01, β_std_ = .48.

Based on a reviewer request, we also conducted an omnibus linear multiple regression to assess whether participant sex, years of experience with the campus, or sense of direction predicted overall angular error (averaged across all four conditions). The overall regression was non-significant, *F*(3, 35) = .86, *p* = .47, *R* ^2^ = .08.

### Experiment 2 Discussion

Our second experiment demonstrated that even without perceiving the environment during testing, participants produced faster responses with lower angular error when default vectors were aligned with road orientations. Aligning with true or magnetic cardinal axes produced angular error rates very similar to aligning with a random, oblique direction. Response times showed a similar pattern. Supporting Experiment 1, individual differences in sense of direction predicted lower angular error, but specifically when participants were aligned with roads or an off-axis orientation. Overall, we found support for our hypothesis of privileged spatial memory access when participants are aligned with road orientations rather than cardinal directions.

## Discussion

Natural and built environments show highly varied alignment between large-scale features (e.g., roads, rows of buildings, mountain ranges) and cardinal directions. In some cases, large-scale features align with cardinal directions and can produce evidence for north-oriented cognitive maps [5,6]. The present results suggest this earlier evidence does not reflect a necessary feature of cognitive maps, but may emerge due to coincidental alignment between environmental features and cardinal directions. In an environment with large-scale features oriented oblique to cardinal direction, two experiments demonstrated no evidence for north-oriented cognitive maps. The first experiment showed generally poor awareness of cardinal direction among pedestrians navigating a familiar campus environment. If cognitive maps were oriented toward north [5,6], pedestrians should show awareness of the north direction while navigating, particularly as spatial memories become better formed with extended experience with the environment [20,21]. Instead, participants showed generally low awareness of cardinal directions and this pattern was found in pedestrians with both low and high levels of experience with the campus environment. Rather than structuring their understanding of the environment based on cardinal direction, participants showed a reliance on road orientation, producing pointing estimates parallel or perpendicular to road axes. In this particular environment, which was oriented oblique to cardinal axes, such a strategy fails to accurately guide cardinal direction estimates. Of course, participants may still be able to orient themselves successfully in the presence of a map, though we did not test this possibility.

### Individual Differences

Our first experiment also demonstrated that pedestrians exhibit individual differences in their ability to orient north, with higher sense of direction scores predicting lower angular error in pointing estimates. As suggested by prior research, skilled navigators may maintain awareness of global spatial references systems and use this information to guide navigation [11,22,23]; these global reference systems may include cardinal direction in some environments, whereas in others (like the one presently used) they are bound to large-scale environmental features. In contrast, navigators with relatively low sense of direction may rely on relatively local cues like individual landmarks and turn directions to guide movement. Still others may show flexible reliance on varied reference systems as they become more or less available, salient, or easy to use [24].

Because roads are highly salient and functional features for navigating pedestrians, there was a chance that Experiment 1’s field data reflected task-relevant reliance on roads [14] rather than the structure of spatial memory. Thus, Experiment 2 required spatial memory retrieval while indoors and unable to directly perceive the campus environment, using a task well-validated in the extant literature. With this task, we found strong evidence supporting reliance on road orientations for structuring spatial memory. When participants were mentally oriented parallel with cardinal directions (true or magnetic), they were markedly slower and more error prone when retrieving relative direction information. In contrast, participants showed the lowest error rates and fastest response times when their imagined orientations were aligned with the road network. This pattern was robust across participants of varied sex and years of experience with campus. Participants with higher sense of direction were generally more accurate at making relative direction judgments, especially when aligned with roads or an off-axis orientation. Together with our first experiment, these data strongly support our hypothesis that north orientation is not a necessary feature of cognitive maps.

### Theoretical Implications

Accuracy in pointing towards north likely relies on: 1) experiential knowledge; 2) map knowledge; 3) ability to align experiential and map knowledge; and 4) the actual alignment between cardinal directions and environmental features. Without access to a map or compass, experiential knowledge of an environment results in learning the directions towards landmarks relative to the body (egocentric), but also directions relative to salient environmental features (allocentric) [25–27]. Spatial memory research demonstrates that individuals assign a “privileged axis” [28] to salient patterns in the environment, such as rows of landmarks [29,30], or their physical facing direction when initially learning the environment [19,31]. In contrast, knowledge gained from a map privileges the top of the map [32,33] and given the prevalence of north-up maps, the privileged direction is typically north.

In the current experiment, the oblique alignment between cardinal directions and road networks around the Tufts campus likely interfered with pedestrians’ ability to align their experiential and map knowledge. The process of aligning cardinal directions with oblique environmental features is likely similar to using a misaligned You-Are-Here map, in that a mental transformation strategy must be chosen and applied correctly [34]. However, unlike using a misaligned map, cognitive maps must be recalled from memory and any transformations must be imagined. It is not currently clear what types of transformation strategies participants attempted to use, or their effectiveness. Mental transformation strategies likely require considerable cognitive load (similar to using a misaligned You-Are-Here map [35]), contributing to pointing errors and a reliance on visual cues over map knowledge. Greater accuracy in pointing towards north, for good sense of direction individuals, might have been due to selecting and/or implementing more accurate transformation strategies.

### Limitations

The Tufts University campus provides a unique opportunity to examine spatial representations of an environment with large-scale environmental features oriented oblique to cardinal direction. We do point out, however, that while the primary campus map viewed by incoming students and visitors (http://campusmaps.tufts.edu/medford/) is aligned toward true north, a printable map available on the campus website is indeed aligned congruent with major road axes (http://campusmaps.tufts.edu/docs/Tufts_Medford-Som_Map.pdf). While it seems unlikely relative to viewing the north-aligned map, it is possible that some of our participants had been exposed to the road-aligned version, altering the orientation of their cognitive maps [6]. We have no direct data to support or refute this possibility. We might expect that if this were the case, however, increased familiarity with the environment should reduce such an influence given a presumably lower reliance on maps; our data do not support such a pattern.

Whereas our first experiment clearly demonstrates value of the SBSOD in predicting pointing error, our second experiment provides relatively equivocal results. Experiment 2 results suggest that higher SBSOD predicts lower angular error in the road-aligned and control conditions, but not the north-aligned conditions (as might be expected [11]). However, this result may be due to a relatively restricted range of SBSOD scores in Experiment 2 which tended to occupy the center of the scale range. A larger or more diverse sample may have afforded a more robust understanding of SBSOD influences on angular error in our JRD task.

## Conclusions

Overall, we demonstrate that navigators spontaneously assign a global reference system to aid in organizing and retrieving memories of familiar environments. This reference system, however, is not always based on cardinal direction. Rather, spatial memory appears to flexibly shift reliance on alternate reference systems based on the (mis)alignment of environmental features with cardinal direction.

## Supporting Information

S1 Dataset Experiment 1 pointing data in absolute degrees relative to magnetic north, including participant age, sex, years of experience with campus, and SBSOD. (XLSX)

S2 Dataset Experiment 2 error rates (columns F-I) and response times (columns J-M) for conditions aligned with magnetic north (“Mag”), true north (“True”), roads (“Road”), or arbitrary orientations (“Ctrl”). Includes also participant age, sex, years of experience with campus, and SBSOD.
(XLSX)
